# Types and Timing of Teaching During Clinical Shifts in an Academic Emergency Department

**DOI:** 10.5811/westjem.2020.10.47959

**Published:** 2021-01-29

**Authors:** Joshua J. Baugh, Derek L. Monette, James K. Takayesu, Ali S. Raja, Brian J. Yun

**Affiliations:** Harvard Medical School, Massachusetts General Hospital, Department of Emergency Medicine, Boston, Massachusetts

## Abstract

**Objectives:**

Academic emergency physicians must find ways to teach residents, medical students, and advanced practice providers amidst the myriad demands on their time during clinical shifts. In this study, we sought to characterize in detail what types of teaching occurred, how often they occurred, and how attending teaching styles differed at one academic emergency department (ED).

**Methods:**

We conducted this observational study in a large, urban, quaternary care, academic Level I trauma center with an emergency medicine (EM) residency. The on-shift activities of EM attending physicians (attendings) were observed and recorded over 42 hours by a fourth-year EM resident with co-observations by an EM education fellow. Teaching categories were identified, developed iteratively, and validated by the study team. We then characterized the distribution of teaching activities during shifts through the coding of attending activities every 30 seconds during observations. Teaching archetypes were then developed through the synthesis of notes taken during observations.

**Results:**

Attendings spent a mean of 25% (standard deviation 7%) of their time engaging in teaching activities during shifts. Of this teaching time 36% consisted of explicit instruction, while the remaining 64% of teaching occurred implicitly through the discussion of cases with learners. The time distribution of on-shift activities varied greatly between attendings, but three archetypes emerged for how attendings coupled patient care and teaching: “in-series”; “in-parallel modeling”; and “in-parallel supervision.”

**Conclusions:**

Teaching in this academic ED took many forms, most of which arose organically from patient care. The majority of on-shift teaching occurred through implicit means, rather than explicit instruction. Attendings also spent their time in markedly different ways and embodied distinct teaching archetypes. The impact of this variability on both educational and patient care outcomes warrants further study.

## INTRODUCTION

Emergency physicians (EP) face myriad demands for their time on shift, including evaluating patients, performing procedures, reviewing charts, communicating with team members, and documenting in the health record. In academic emergency departments (ED), responsibilities for part of these tasks are shifted to residents, medical students, and advanced practice providers. However, attendings in academic departments are also expected to teach and supervise, creating different and additional demands for their time. Prior research suggests that attendings work just as quickly when paired with medical students and may actually see more patients per hour when working with residents.^1^–^4^ As technology – particularly electronic health records – re-shapes how physicians practice,^5^ it is important to understand how on-shift teaching fits into the other activities expected of today’s academic EP.

Prior studies have described the proportion of time that physicians spend on patient care, documentation, and other activities in both community and academic EDs. Academic attendings have been reported to spend anywhere from 6–20% of their shifts teaching.^6^,^7^ However, the definition of teaching in these studies has either been limited in description or defined as all conversations between attendings and learners. While the raw amount of time attendings spend with residents does appear to affect resident learning experience on shift,^8^,^9^ this cannot capture the complex nature of on-shift education in the ED. Indeed, widely varying methods by which expert attendings teach during busy shifts have been described.^10^,^11^ The optimal mode of education in chaotic emergency medicine (EM) environments remains unknown; a more nuanced account of how teaching currently occurs in academic EDs is needed to lay the groundwork for the future study of educational efficacy during shifts.

In this study, we sought to characterize in detail how teaching occurred in one academic ED. We aimed to describe the different forms of teaching that took place during shifts, as well as how teaching interacted with the other activities expected of academic attending physicians. We set out to do this through detailed observations of attending physicians involving quantitative assessments of activity frequencies and qualitative development of teaching-style archetypes.

## METHODS

### Setting

This observational study was conducted in a large, urban, quaternary care, academic Level I trauma center, with an EM residency. It was considered quality improvement by the institutional review board and therefore exempt from review. All observations were conducted in the 25-bed critical care area of the ED. This area sees an average of 52 patients per day; 60% of these patients are admitted. The area is supervised by one attending EP at all times of day, with varying levels of staffing by residents and physician assistants depending on time of day. There is always at least one senior resident working in this area, and there is usually at least one junior resident as well. The levels of additional trainees on shift vary from intern to senior resident; there is nearly always a mix of trainee levels present, but the particular combination changes day to day.

### Data Collection

The primary mode of data collection was direct observations of attending physicians during shifts. The primary observer was a fourth-year EM resident. A total of 42 clinical hours were observed in 10 four-hour blocks and one two-hour block. All observed shifts took place on weekdays, and observation blocks were either 10 am-2 pm or 3 pm-7 pm (plus one 12-2 pm block), with a near-even distribution between the times. These periods were chosen because they generally have high patient arrival rates, exemplifying the need for attendings to use their time thoughtfully. One attending was observed in each period, and 10 different attendings were observed over the course of the 11 observation blocks (one attending was observed twice).

**Population Health Research Capsule**

What do we already know about this issue?

Academic emergency physicians (EP) use many techniques to teach during shifts and must balance teaching with other tasks.

What was the research question?

How much do academic EPs teach during shifts, what types of teaching occur, and how is teaching paired with clinical care?

What was the major finding of the study?

Attendings devoted 25% of their time during shifts to teaching, most of which was implicit through case discussion. Three teaching archetypes were identified.

How does this improve population health?

Effective on-shift teaching is essential for training the next generation of EPs. Understanding when and how teaching occurs can help to improve trainee education.

At the outset of each observation, the primary observer obtained verbal assent from each attending. During assent, attendings were told that their activities would be observed and recorded in writing. Attendings were not informed of the study goals and did not know that educational activities were an outcome of interest. The observer then recorded the activities of the attendings in 30-second increments by writing down each observed activity on a paper template with a line for every 30-second period. If more than one activity occurred in a 30-second period, the dominant activity was recorded. For activities involving an interaction with a learner, the role of the learner was also recorded (learners were defined as either medical students, residents, or physician assistants in this study).

### Defining Activity Categories

We categorized attending activities into teaching and non-teaching-related subcategories. The activity categories were developed iteratively over the course of two observation periods; an initial coding scheme was created based on presupposed categories, and then modified based on actual observed activities. These coding categories were further refined through iterative discussion by study team members (all of whom are EPs), and then validated through observation by a second observer.

### Assessing Inter-rater Reliability

The second observer (an EM education fellow) joined the primary observer for two two-hour co-observations sessions to validate the activity codes. During these sessions, each observer independently recorded the activities observed every 30 seconds, choosing from among the previously agreed-upon categories and definitions. Subsequently, observations were compared by 30-second increment, and a Cohen’s kappa statistic was calculated to assess inter-rater reliability.

### Developing Archetypes with Flow Diagrams

In addition to assigning activity categories, the primary observer also took notes during observations on the teaching styles of the observed attendings. During observations it became apparent that different attendings used distinct strategies for coupling teaching and patient care. Using field notes, the primary observer identified three archetypes that encompassed most attending teaching behavior. These archetypes were refined with the second observer and then with the study team. Flow diagrams were created to demonstrate the pattern of teaching and patient care observed with each archetype.

### Outcomes

The primary outcome was the percentage of time attendings spent in total on the various types of teaching during shifts. The average amount of time spent by attendings on each specific type of teaching was characterized using the teaching categories and coding strategy described above. We also calculated the amount of time spent on other activity categories by similar means. In addition, a box plot was created in the open source statistical program R Studio v1.2.5001 (Boston, MA) to demonstrate the variability in activity distribution across the attending physicians observed. We also assessed the amount of time spent teaching residents vs physician assistants by calculating and comparing the amount of observed teaching time conducted with each type of learner.

## RESULTS

### Inter-rater Reliability

The two observers achieved a kappa of 0.89. Inter-rater agreement was 90% among the 30-second increments that at least one observer labeled as a teaching activity. Among instances of disagreement, 43% were due to disagreement over the start and end points for an activity, as opposed to how the activity was coded. In 20% of disagreements observers concurred that teaching was occurring, but they disagreed on the teaching subtype. Finally, 37% of disagreements involved the observers coding 30-second increments as entirely different activity categories; 75% of these involved one observer coding an action as a teaching activity when the other observer did not.

### Primary Outcomes

Overall, teaching activities comprised a mean of 25% (standard deviation [SD] 7%) of attendings’ time during the observed periods of this study. We identified two principal categories of teaching: explicit and implicit, with 36% of total teaching time categorized as explicit, and 64% implicit. Implicit teaching occurred through back-and-forth discussions of patient cases with learners but did not involve the attending expressly providing new medical information in a didactic teaching format to the learner, nor clear instruction in how they would proceed in a given case. Often this kind of teaching consisted of asking questions or exploring alternative diagnoses and was observed to blend in with the management of patient care. Explicit teaching occurred when the attending clearly made education the main intent of their words or actions, eg, providing novel information from a recent study to the learner, describing their own personal approach to a difficult situation, or instructing the learner in how to perform a procedure. This explicit teaching was generally observed to be identifiably separate from the routine management of patient care.

Within explicit teaching, four subcategories were identified: case-based teaching; procedural teaching; bedside teaching; and topic-based teaching. Final teaching-related and non-teaching activity subcategories are displayed in Tables 1 and 2, respectively. Case-based teaching was the most common, comprising 52% of explicit teaching. Topic-based teaching was relatively rare, comprising 7% of explicit teaching. See Table 3 for further time breakdown by explicit teaching category.

**Figure 1:**
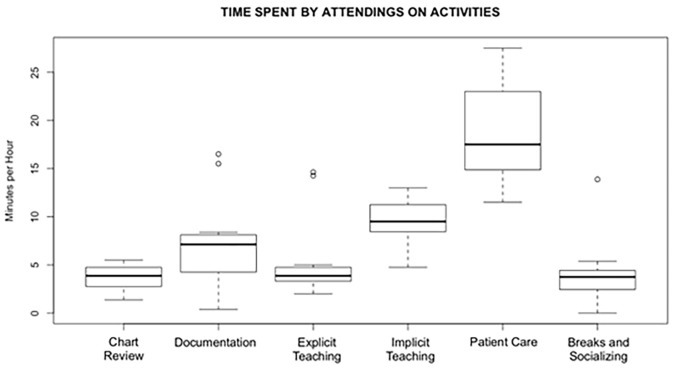
Box plot for the distribution of on-shift activities across observations. Bold lines represent median values, solid boxes delineate 25th–75th percentiles, dotted lines delineate the full data range, and circles represent outliers. N = 11.

While the above statistics represent averages, marked variability was observed in the amount of time that different attending physicians spent on teaching activities and all other activities; explicit teaching constituted anywhere from 3–24% of time on shift, while total teaching (implicit and explicit combined) ranged from 17–40% of on-shift time. See Figure 1 and Table 4 for graphical and numerical depictions of activity variability by attending.

### Teaching Archetypes

**Figure 2:**
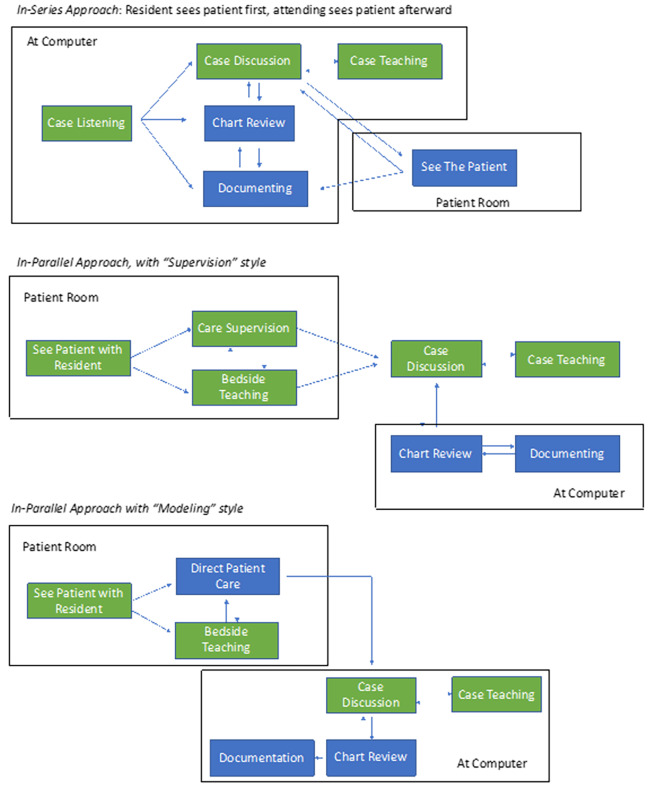
Flow diagrams for the three archetypes for how attending physicians see patients and incorporate education. Blue boxes delineate solo activities; green boxes delineate teaching activities.

Three main archetypes emerged for how attendings coupled patient care and education, with flow diagrams of each depicted in Figure 2. While no attending used exclusively one archetype throughout an observation period, all of them had a dominant style that they employed most of the time. We labeled the three identified archetypes as “in-series,” “in-parallel with supervision,” and “in-parallel with modeling.” The difference between in-series and in-parallel was whether attendings saw patients separately from learners (in-series) or simultaneously with learners (in-parallel).

“In-series” describes a style where attendings let the learner see a patient first, received a presentation on the case, and then saw the patient separately. This approach often led to implicit teaching during the presentation, followed sometimes by explicit teaching once the attending had seen the patient. This “in-series” style was used in about 40% of total patient encounters throughout the observations, and three of the attendings observed displayed this archetype predominantly.

“In-parallel” approaches were used in approximately 60% of patient encounters and could be enacted in a “supervision style” or a “modeling style.” Supervision involved quietly observing as the learner engaged with the patient, interjecting only occasionally as needed to ensure adequate clinical care. Modeling involved the attending engaging directly with the patient and executing most of the history and physical while the learner observed. The in-parallel style was much more likely to involve bedside teaching than the in-series style. Four attendings primarily used in-parallel supervision, while three attendings primarily used in-parallel modeling.

### Time with Residents vs Physician Assistants

On average, residents spent 3.1 minutes per hour receiving any type of teaching from an attending, while physician assistants spent 2.3 minutes receiving any type of teaching from an attending. Of note, while residents and physician assistants were staffed relatively evenly over the observed time periods (55% residents, 45% physician assistants), over 75% of explicit teaching time was directed toward residents.

### Other Activities

Across observations, attendings spent a mean of 32% (SD 9%) of their time on direct patient care, 12% (SD 8%) on documentation, 7% (SD 6%) socializing or taking breaks, and 6% (SD 2%) on chart review (Table 5). Attendings saw a median of 2.9 (SD 0.59) patients per hour and spent a median of 5.8 (SD 2.6) minutes in each new patient’s room.

## DISCUSSION

In this academic ED, attending physicians spent 25% of their time on activities involving teaching, with 9% of total time spent on explicit teaching. When explicit teaching occurred, it was most often case-based, followed by bedside and procedural teaching; formal topic-based teaching was rare in our setting. The majority of teaching was implicit, with important lessons transmitted through questions asked and discussions initiated in the normal flow of managing patient care. These conversations did not involve the explicit didactic transmission of new knowledge, but they did provide learners with opportunities to observe how attendings thought about cases, what information attendings found most pertinent, and other implicit features of how attendings approached their work as EPs.

While it was clear to the observers that implicit teaching held potential educational value for learners, it is not known whether attendings or learners experienced these interactions as “teaching.” Studies evaluating the aspects of clinical teaching most valued by residents suggest that these characteristics will evolve over the course of residency.^12^,^13^ Previous clinical experience may therefore influence which archetypes learners perceive to be teaching vs supervision without educational value. Our study was not designed to elucidate this nuance, but it will be important for future work to assess perceptions of both attendings and learners about the types of education that occur during shifts.

Our data show there are many ways to structure an attending’s time during an academic ED shift; consistent with prior research on EP tasks, there was marked variability in the distribution of both teaching and non-teaching activities between attendings in our study.^14^ However, we know relatively little about how the mix of activities chosen by attendings may affect educational quality, documentation quality, and perhaps even patient care quality. At minimum, prior work suggests that the need to manage multiple ED patients in a short period has an impact on bedside teaching, an important part of the “in-parallel” archetype.^15^ Future research might further explore the impact on various outcomes of how academic EPs spend their time during shifts.

Attendings also differed in their approach to integrating education and patient care, which in turn affected the types of teaching that occurred. Attendings and learners saw patients either “in parallel” or “in series,” with two versions of “in parallel” observed: “supervision” and “modeling.” While the level of the learner and the nature of the case may have affected the style chosen, it became clear that most attendings gravitated to one archetype regardless of other factors. Prior research does suggest that as the acuity of patient cases rises, attending’s choice of educational strategy narrows;^16^ it is, therefore, possible that our critical care area setting influenced the pattern of archetypes we observed.

The archetype embodied by an attending likely affects the experience of the learner, the experience of the patient, and the experience of the attending in providing care. With the “modeling” strategy, learners may benefit from observing how attendings interact with patients but miss out on opportunities for developing autonomy. With this style, the attending can engage with patients themselves, and patients receive care directly from the attending. “Supervision” allows the learner to practice their patient care skills directly and in the best-case scenario, provides an opportunity for the attending to give the learner feedback and conduct resident milestone assessments required by the Accreditation Council for Graduate Medical Education.^17^ However, the attending engages minimally with the patient directly in this style, and the patient interacts mostly with a learner. “In-series” patient care allows learners more independence, which may have its own educational advantages. The attending is also able to engage with the patient directly, but patients must tell their story twice. Each style therefore likely has advantages and disadvantages for education, attending experience, and patient experience. A recent study on “swarming,” which maps to the “in-parallel” archetypes here, suggests the practice can have efficiency and educational benefits in a pediatric ED.^18^ The impact may be different in the adult ED environment, and the way an attending leads the “swarm” likely matters. Future research might examine the relative effects of the archetypes we observed on educational and patient care outcomes.

## LIMITATIONS

This was a single institution study. Observations occurred only on weekdays at specific times in an acute care area of the ED; time spent on teaching and other activities may have varied at different times of day, on weekends, and with different mixes of cases. Our results were likely affected by the particular ratio of attending to learners in our setting, as well as expectations around patient volumes seen by our attendings; this may limit generalizability to other settings with different staffing structures and expectations. While we observed varied teaching methods, we were not able to assess teaching effectiveness or how learners perceived the educational value of different methods. We were also unable to assess why attendings structured their time in the different ways that we observed.

## CONCLUSION

Attending physicians in this academic ED spent a quarter of their time teaching, most of it through implicit means. Attendings varied widely in how they spent time during shifts but fit into three distinct archetypes of how education was structured in relation to patient care. Future research should examine the impact of these choices and archetypes on educational and patient-related outcomes.

## Figures and Tables

**Table 1:** Activity categories recorded for attending interactions with learners.

Implicit teaching:
Case discussion: The attending actively engages the learner in discussion about a case with back-and-forth conversation, but without didactically imparting new knowledge or providing clear instruction on topics related to a case.
Explicit teaching:
Case-based teaching: The attending provides clear didactic instruction using content of a case, novel information pertaining to a case, or a description of how they would personally handle a case.
Bedside teaching: The attending provides clear educational instruction to the learner at the patient’s bedside using the content of the patient’s presentation and/or physical exam findings.
Procedural teaching: The attending directly teaches and supervises a learner in how to perform a bedside procedure, including ultrasound.
Topic-based teaching: The attending provides formal didactic education to a learner about a topic not related to a case seen with that learner.
Not considered teaching:
Case listening: The attending purely listens to a case presentation from a learner without giving any input.

**Table 2:** Activity categories recorded for attending activities not involving learners.

Documenting: Attending inputs data into the medical chart or dictates to a scribe.
Chart review: Attending looks at patient data on the computer or asks a scribe to read data from computer.
Initial patient care: The first time the attending enters the patient room. (It was also documented whether the attending saw patients alongside residents or independently.)
Re-evaluation patient care: Subsequent times the attending enters a patient room.
Emergency medical services’ (EMS) report: Attending listens to EMS calls.
Break: Attending takes personal time, including using personal phone, e-mail, eating, using restroom.
Socializing: Attending speaks with team members about topics unrelated to medical care.
Sign-out: Attending takes sign-out from oncoming team.
Walking: Attending walks between ED locations while not engaged in another activity.
Phone call: Attending is on the phone with consultants or other hospital staff.
Team communication: Attending speaks with members of the team other than residents or advanced practice providers (eg, nurses, techs, pharmacists).

*ED*, emergency department.

**Table 3:** Percentage of total explicit teaching time spent on different teaching subcategories.

Explicit teaching category	Percentage
Case-based	52%
Bedside	22%
Procedural	18%
Topic-based	7%

**Table 4:** Variability in how attendings spent time on shift, with the range between extremes for each activity category.

Activity	Range of minutes/hour
Explicit teaching	2–15
Implicit teaching	7.8–19
Direct patient care	11.5–25
Documentation	1–16.5
Chart/Data review	1.3–5.5
Breaks/Socializing	0–13.8

**Table 5:** Activity distribution for on-shift activities. No standard deviation listed for “Other” as the relative composition of this category varied between observations.

Activity	Avg min per hour (SD)	Percentage
Explicit teaching	5.5 (4.4)	9%
Implicit teaching	9.6 (2.3)	16%
Direct patient care	18.9 (5.6)	32%
Documentation	7.5 (5)	12%
Chart/Data review	3.6 (1.3)	6%
Receiving sign-out	3.6 (1.5)	6%
Breaks/Socializing	4.2 (3.6)	7%
Other (EMS calls, case listening, phone calls, speaking with consultants, walking, team communication)	6	10%

*Avg*, average; *min*, minute; *SD*, standard deviation; *EMS*, emergency medical services.
